# The complete mitogenome of *Paralonchurus dumerilii* (Perciformes: Sciaenidae)

**DOI:** 10.1080/23802359.2020.1840932

**Published:** 2022-06-08

**Authors:** Ana Carla Barros de Souza, Jeferson Carneiro, Arturo Angulo, Rafael Ribeiro Barata, Márcio Nunes, Iracilda Sampaio

**Affiliations:** a Instituto de Estudos Costeiros, Universidade Federal do Para, Bragança, Brazil; b Museo de Zoología y Centro de Investigacion en Ciencias del Mar y Limnologia (CIMAR), Universidad Costa Rica, San Jose, Costa Rica; c Instituto Evandro Chagas, Ananindeua, Brazil

**Keywords:** mtDNA, phylogeny, *Paralonchurus dumerilii*, fish, Perciformes, Eastern Pacific Ocean

## Abstract

We herein describe the complete mitochondrial genome of *Paralonchurus dumerilii* (Sciaenidae) and infer its phylogenetic position within the family. The genome is 16,498 bp long and featured by 13 protein-coding genes (PCGs), two rRNAs, 22 tRNAs, and a control region (D-loop). Our phylogenetic analysis suggests a basal position of *P. dumerilii* as the sister group of the other species of Sciaenidae analyzed.

The suco croaker or striped corvine, *Paralonchurus dumerilii* (Peciformes, Sciaenidae), occurs in coastal water on the east coast of America, from Guatemala to Chile, and it is mainly captured by trammel nets as accompanying fauna (Soto et al. 2012). Tissue (muscle) samples from a specimen of *P. dumerilii*, collected in Costa Rica and deposited at the fish collection of the Universidad Costa Rica (CTP-1197), were obtained and preserved in 96% ethanol. The genomic DNA was extracted from the sample using a Wizard Genomic DNA Purification Kit (Promega, Madison, WI), according to the manufacturer instructions, and sequenced using the shotgun sequencing method on the Illumina 2500 Hiseq platform. The obtained reads were assembled with IDBA-UD and used 21–99 km (Peng et al. 2012). A sequence of 16,498 bp (GenBank accession number: MT904198) was obtained and annotated on the Mitos platform (Bernt et al. 2013); it was tRNA reevaluated in tRNAscan-SE1.21 (Lowe and Eddy 1997) and, curated manually using the complete mitotic genome sequence of the Sciaenidae contained in the NCBI database. Then, an analysis of relative synonymous codon usage (RSCU) for the 13 protein-coding genes (PCGs) was performed on MEGA version 7 (Kumar et al. 2016). A phylogenetic tree, based on maximum likelihood and RAxMLGUI (Silvestro and Michalak 2012) using the 13 PCGs, was generated. The complete mitochondrial genome of *P. dumerilii* is 16,498 bp long, the gene order and transcriptional direction occur as typical in vertebrates, including 13 PCGs, two rRNAs, 22 tRNA, and an 819 bp non-coding control region (D -loop). The global nucleotide composition is: 27.42% A, 30.83% C, 16.18% G, and 25.57% T. The 12S (951 bp) and 16S (1711 bp) *rRNA* genes are located between tRNA-Phe and tRNA-Leu and separated by tRNA-Val, the tRNA lengths varied from 68 to 74 bp. The 13 PCGs from *P. dumerilii* are conserved in relation to the other species of Sciaenidae and ranged from 168 bp (atp8) to 1839 bp (nd5). Among the PCGs were observed a single start codon (ATG) and several distinct stop codons, while some of them are incomplete with a T or TA termination, being completed by a post-transcriptional polyadenylation. The RSCU analyzes revealed that the codons L (CUC), L (CUU), P (CCC), and P (CCU) are more frequent, while the codons A (GCG), D (GAU), V (GUG), and R (CGU) are relatively rare. Six genes are overlapping, and the length of the overlap varies from 4 to 10 nucleotides. The phylogenetic analysis recovered *P. dumerilii* as the sister group of the other species of Sciaenidae analyzed in this study (Figure 1). Altogether the results contribute to elucidate the evolutionary history of the family.

**Figure 1.**
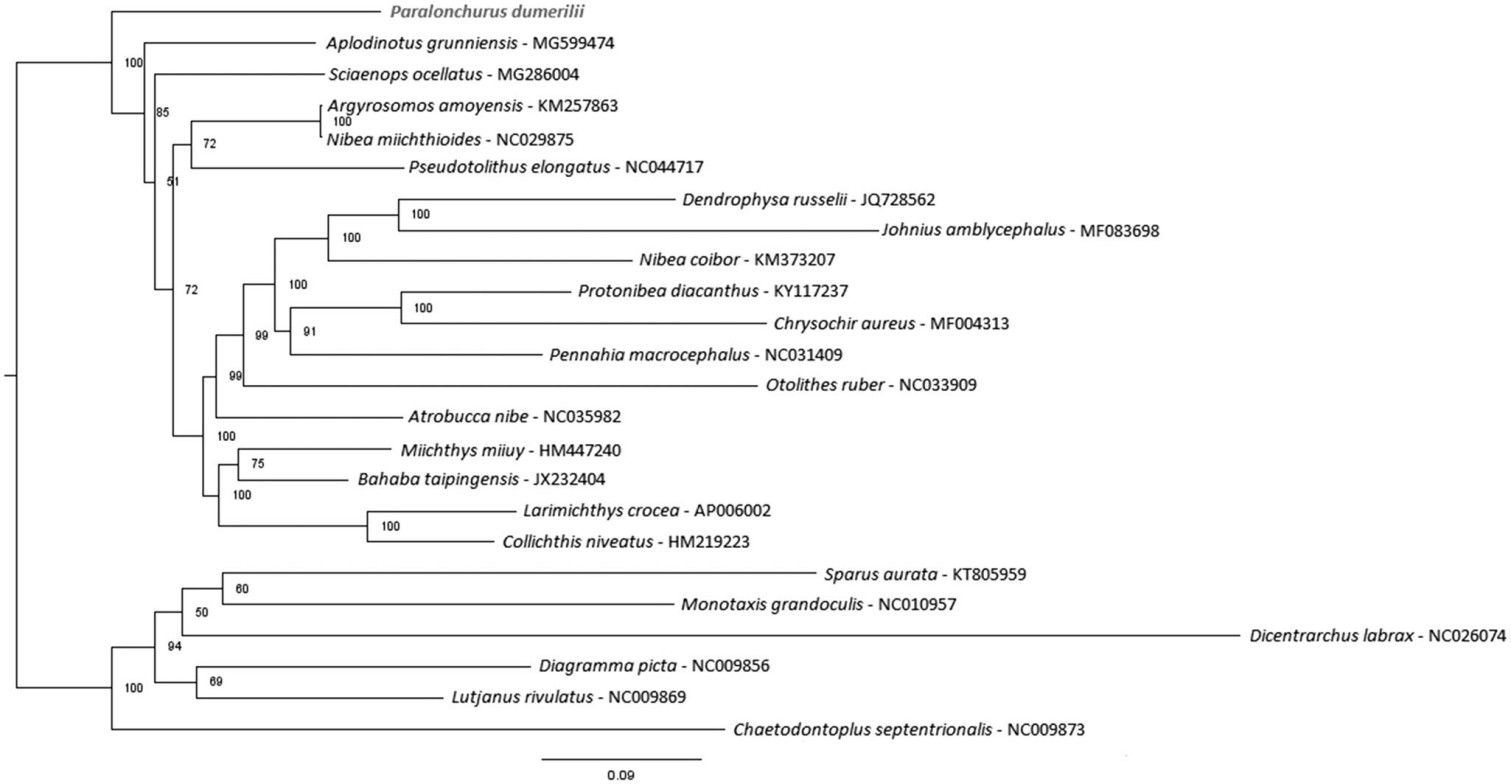
The topology of the Sciaenidae phylogenetic tree based on the complete mitogenome of *P. dumerilii*. The phylogenetic position of *P. dumerilii* is shown. The topology was inferred from maximum-likelihood analyses. Numbers at nodes represent bootstrap values in percentage. Values below 70% are not shown. The tree is rooted with *Chaetodontoplus septentrionalis*, *Sparus aurata*, *Monotaxis grandoculis*, *Dicentrarchus labrax*, *Diagramma picta*, and *Lutjanus rivulatus*.

## Data Availability

The authors confirm that the data supporting the findings of this study are available within the article and its supplementary materials. The data that support the findings of this study are available in GenBank at www.ncbi.nlm.nih.gov, reference number: MT904198.
